# Author Correction: Mechanochemical tuning of a kinesin motor essential for malaria parasite transmission

**DOI:** 10.1038/s41467-023-36854-w

**Published:** 2023-02-27

**Authors:** Tianyang Liu, Fiona Shilliday, Alexander D. Cook, Mohammad Zeeshan, Declan Brady, Rita Tewari, Colin J. Sutherland, Anthony J. Roberts, Carolyn A. Moores

**Affiliations:** 1 Institute of Structural and Molecular Biology, Birkbeck College, London, WC1E 7HX UK; 2 School of Life Sciences, University of Nottingham, Nottingham, NG7 2UH UK; 3 Department of Infection Biology, Faculty of Infectious & Tropical Diseases, London School of Hygiene & Tropical Medicine, Keppel Street, London, WC1E 7HT UK; 4 Present Address: Department of Biochemistry, University of Oxford, Oxford, OX1 3QU UK

**Keywords:** Cryoelectron microscopy, Parasite biology, Cytoskeletal proteins

Correction to: *Nature Communications* 10.1038/s41467-022-34710-x, published online 16 November 2022

The original version of this Article contained an error in Fig. 4, in which in panel (b) the residues in the sequence presented were highlighted with a wrong color coding, i.e., the second “S” in the sequence “NALVSRSKGTSKSNFIPFRDSKLTR” was highlighted in pink instead of black. In addition, the arrows in panel (a) have decreased thickness to improve the visibility of the structure. This has been corrected in both the PDF and HTML versions of the Article.

