# Supplementary material for: Mechanochemical tuning of a kinesin motor essential for malaria parasite transmission
Source: Nat Commun. 2022 Nov 16;13:6988. doi: 10.1038/s41467-022-34710-x (PMC9669022; doi:10.1038/s41467-022-34710-x)
Supplement: Supplementary file 1 — Supplementary Information [file 41467_2022_34710_MOESM1_ESM.pdf]

Mechanochemical tuning of a kinesin motor essential for malaria parasite transmission

SUPPLEMENTARY INFORMATION

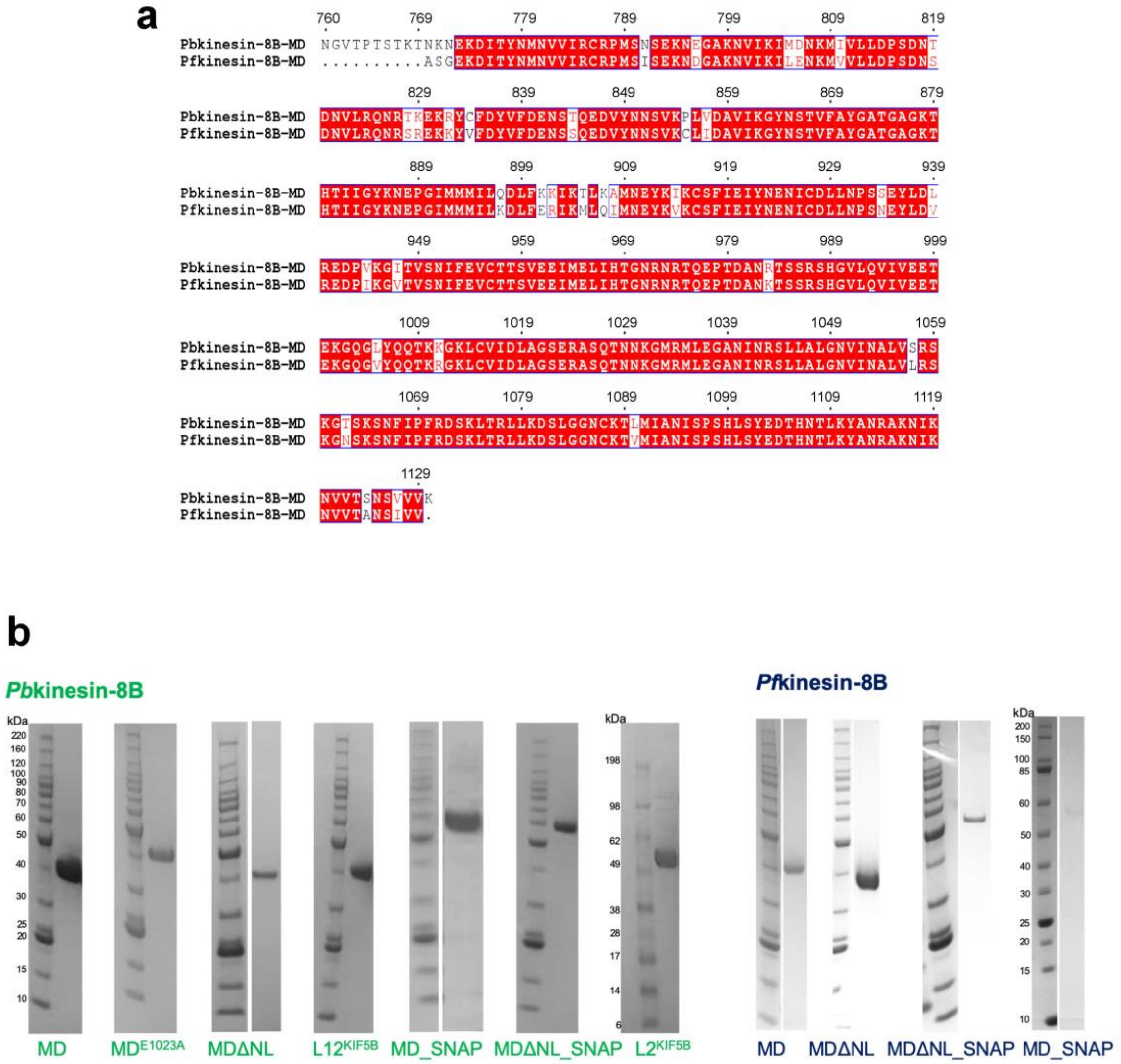

Supplementary Figure 1. Sequence alignment of *Pb*kinesin-8B-MD with *Pf*kinesin-8B-MD and purified kinesin-8B proteins.

a) EMBOSS needle alignment shows 88% identity (shaded red) and 94% similarity (red text) between *Pb*kinesin-8B-MD with *Pf*kinesin-8B-MD protein sequences.

b) SDS-PAGE Coomassie stained gels showing pure proteins used in our experiments. BenchMark™ Protein Ladder (Invitrogen) was used for all gels apart from the one for *Pf*kinesin-8B-MD-SNAP (unstained Protein Standard (Broad Range, NEB) was used) and the one for *Pb*kinesin-8B-MD-L2^{KIF5B}(Invitrogen^{TM} SeeBlue™ Plus2 Pre-stained Protein Standard was used). Empty space between sample lane and marker indicates these 2 lanes are from the same gel but not next to each other. For most constructs, similar protein quantity and quality were obtained from 2 independent purification batches.

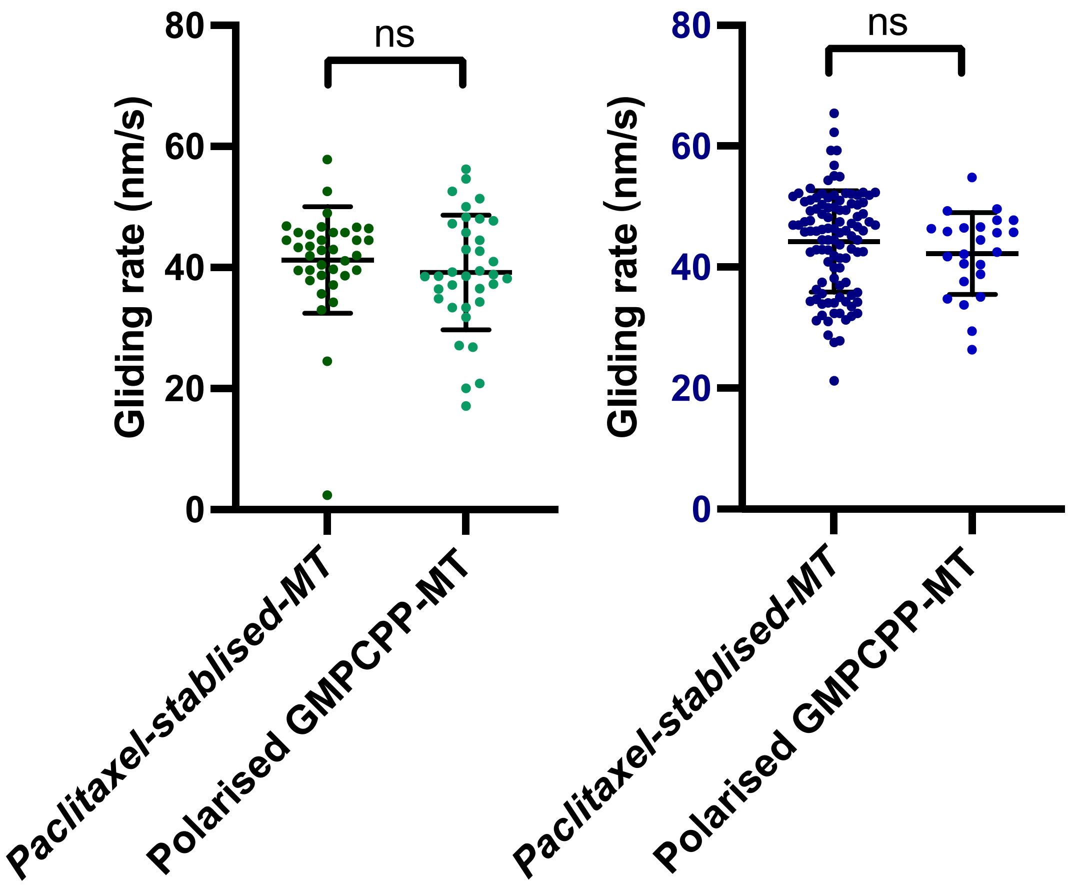

Supplementary Figure 2. MT gliding velocity driven by *Plasmodium* kinesin-8Bs is unaffected by MT stabilisation mode.

No difference in gliding velocity was observed between the non-polarised GDP-paclitaxel MTs and polarised GMPCPP-MTs for both *Pb*kinesin-8B-MD (left) and *Pf*kinesin-8B-MD (right). For *Pb*kinesin-8B-MD, the velocity of GDP-paclitaxel MT gliding = 41.3 ± 8.8 nm/s (mean ± SD; n = 36; data from Fig. 1c replotted here from comparison), the velocity of GMPCPP MT gliding = 39.2 ± 9.5 nm/s (mean ± SD; n = 35). ns, not significant, p = 0.3425 by two tailed t-test. For Pfkinesin-8B-MD, the velocity of GDP-paclitaxel MT gliding = 44.3 ± 8.4 nm/s (mean ± SD; n = 104; data from Fig. 1c replotted here from comparison), the velocity of GMPCPP MT gliding =42.3 ± 6.8 nm/s(mean ± SD; n = 24). ns, not significant, p = 0.2738 by two tailed t-test.

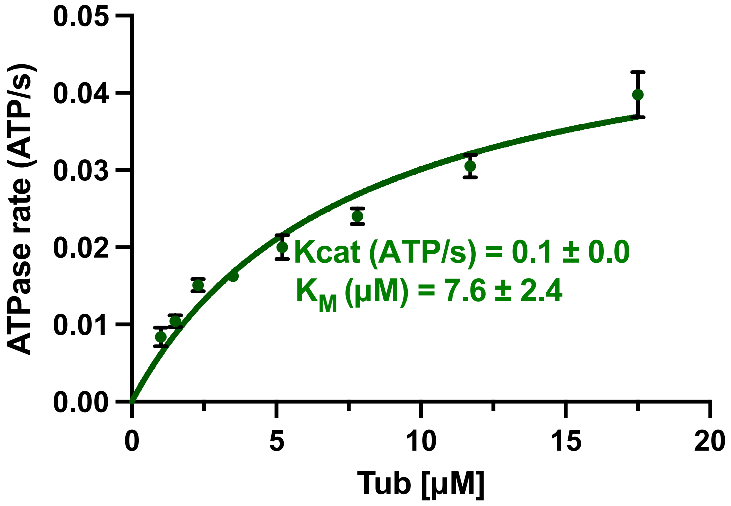

Supplementary Figure 3. ***Pb*****kinesin-8B-MD exhibits tubulin stimulated ATPase activity.** The ATPase assay data (n=3 for each point, mean ± SD) were fitted using Michaelis-Menten equation, from which the Kcat and K_M were calculated in Prism9.

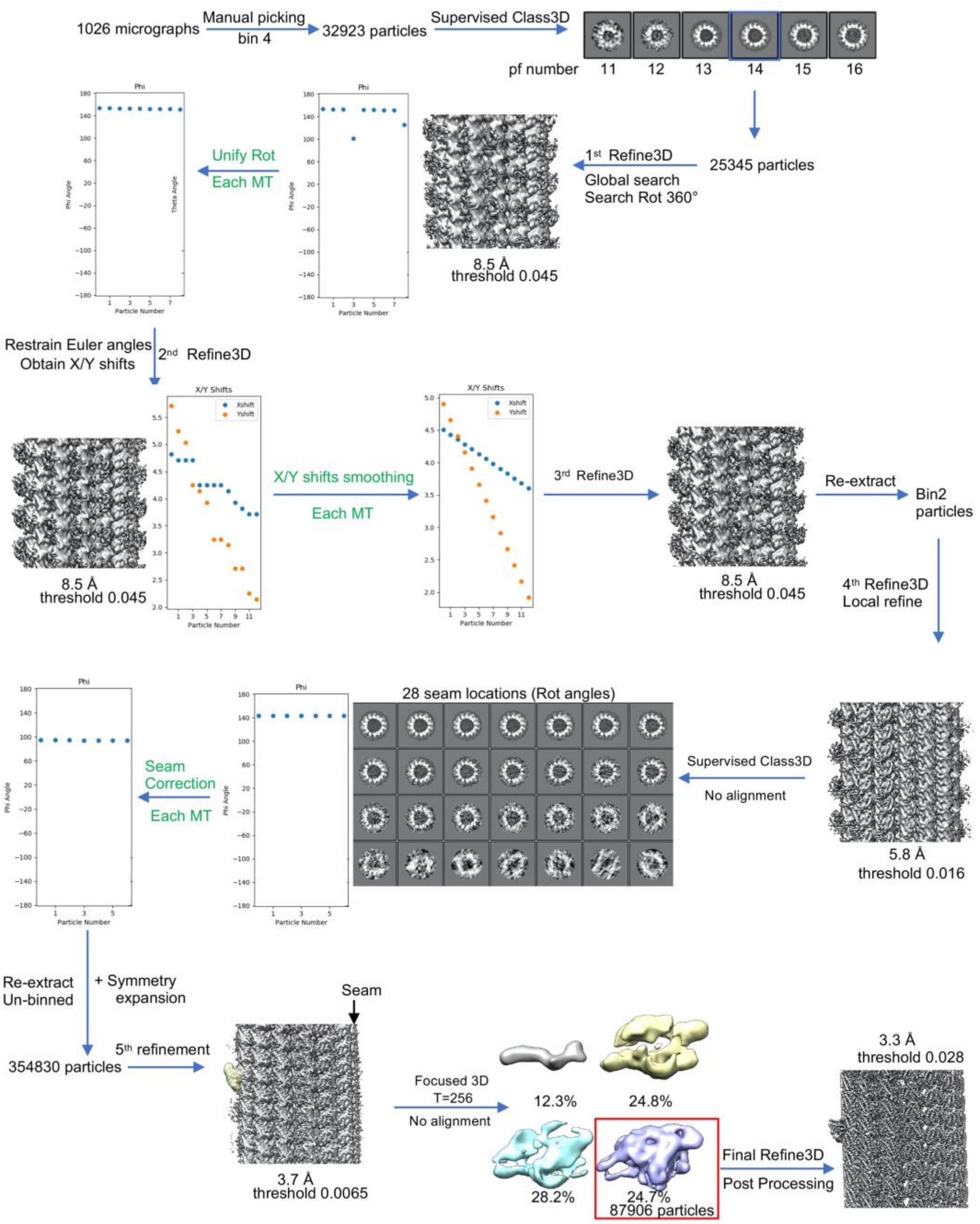

Supplementary Figure 4. 3D image processing workflow for kinesin-bound MT datasets. MT-bound AMPPNP *Pb*kinesin-8B-MD dataset is used as an example. RELION operations are in black and custom MT operation using python scripts in green[1]. The same approach was used for the NN *Pb*kinesin-8B-MD and *Pf*kinesin-8B-MD datasets. Data processing was performed using the MiRP pipeline[2]. Briefly, 4x binned manual picked particles were subjected to supervised 3D classification with 15 Å low-pass filtered references of MTs with different protofilament numbers, and particles from 14 protofilament MTs were pooled for further processing. A global search was performed, where one iteration of 3D alignment with restrained Psi and Tilt and a complete search of the Rot angle and X/Y shifts was performed. For each MT, the most commonly assigned Rot angle from the global search step for each MT was imposed on all particles in that MT. A second round of 3D alignment was performed with restrained Psi, Tilt and Rot and a wide search of X/Y shifts, followed by a custom

operation using a python script to ensure the X/Y shifts of particles from a given MT follow the same slope and intercept. Another refinement with a reduced X/Y shift search area and restricted Phi/Psi/and Tilt angles was done. Then, bin2 particles were re-extracted followed by local refinement. To check each MT Rot angle aligned the seam of experimental images with the seam of the reference, supervised 3D classification without alignment was performed. 28 (14*2) references were synthesised representing all possible seam positions with or without a 41 Å shift along the helical axis. The most common class for each MT was assigned to particles from the same MT and corresponding rotations and translations along the helical axis were then adjusted to ensure the seam of experimental images matched the seam of the reference. C1 reconstruction was performed on un-binned particles using auto-refine with alignment parameters obtained from the above processing steps. To improve the resolution of the kinesin density, a focused 3D classification step was performed following symmetry expansion to select the subset of particles with the highest kinesin occupancy for final averaging.

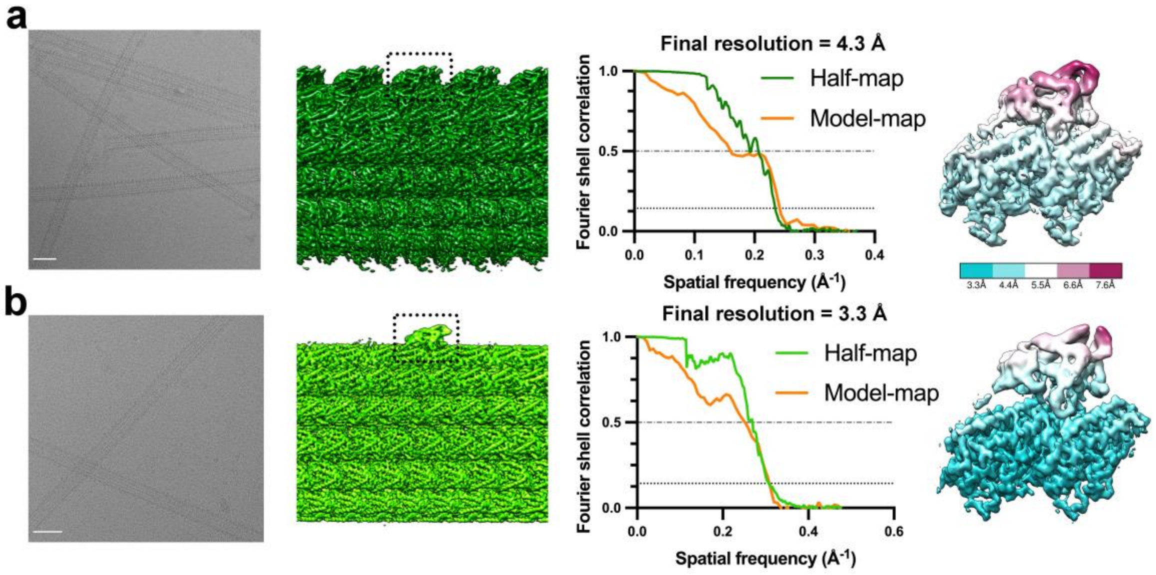

Supplementary Figure 5. Cryo-EM reconstruction resolution estimation.

a) Left, representative digital micrograph of *Pb*kinesin-8B-MD bound GMPCPP-MT in the absence of nucleotide (from 329 micrograph dataset); scale bar = 50 nm; middle left, final reconstruction; data processing details are described in the Methods; middle right: gold-standard FSC curves between the two half maps with indicated resolution at FSC = 0.143 and FSC curves between the atomic model and the final map with indicated resolution at FSC = 0.5 are shown; right, local resolution for best kinesin motor-tubulin dimer.

b) Left, representative digital micrograph of *Pb*kinesin-8B-MD bound GMPCPP-MT in the presence of AMPPNP (from 1,026 micrograph dataset); scale bar = 50 nm; middle left, final reconstruction following 3D classification to optimise motor domain density; in this case, the central motor-tubulin dimer asymmetric unit is enriched; data processing details are described in the Methods; middle right, gold-standard FSC curves between the two half maps with indicated resolution at FSC = 0.143 and FSC curves between the atomic model and the final map with indicated resolution at FSC = 0.5 are shown; right, local resolution for best kinesin motor-tubulin dimer.

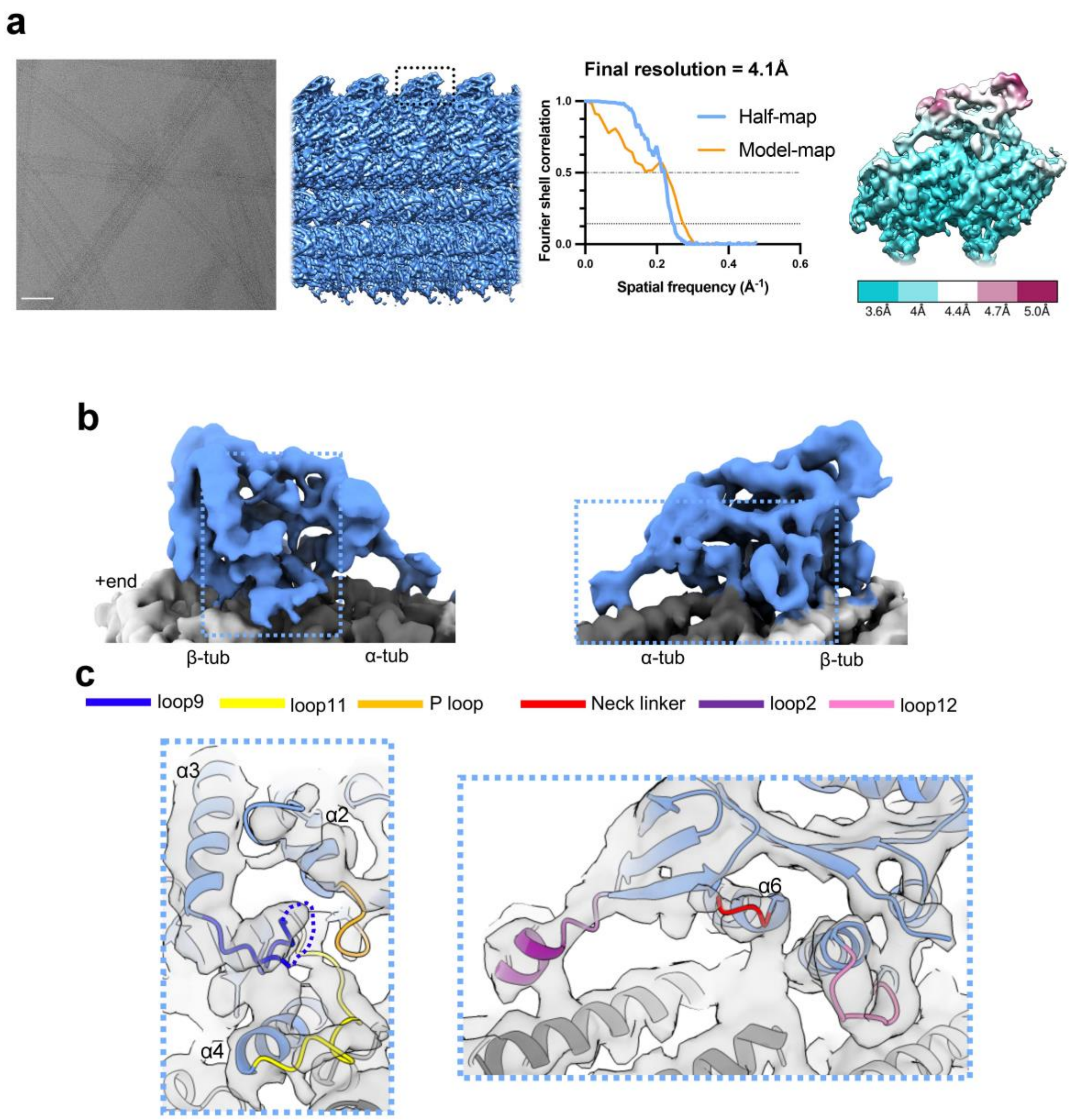

Supplementary Figure 6. Cryo-EM reconstruction of MT-bound *Pf*kinesin-8B-MD NN and resolution estimation.

a) Left, representative digital micrograph of *Pf*kinesin-8B-MD bound GMPCPP-MT in the absence of nucleotide (from 4,075 micrograph dataset); scale bar = 50 nm; middle left: final reconstruction following 3D classification; middle right, gold-standard FSC curves between the two half maps with indicated resolution at FSC = 0.143 and FSC curves between the atomic model and the final map with indicated resolution at FSC = 0.5 are shown; right, local resolution density for best kinesin motor-tubulin dimer.

b) Asymmetric unit of GMPCPP-MT-bound NN *Pf*kinesin-8B-MD depicted as solid surface representation and viewed towards the NBS (left) and the neck linker region (right) at threshold 0.018. *Pf*kinesin-8B-MD-NN density is coloured in blue, α- and β-tubulin are coloured in dark and light grey, respectively; regions around NBS and neck linker depicted in (c) are boxed.

c) Left, zoom-in view of the NBS of NN *Pf*kinesin-8B-MD with docked model; right: zoom-in view of the neck linker region of NN *Pf*kinesin-8B-MD with docked model. *Pf*kinesin-8B-MD-NN model is coloured in blue and α- and β-tubulin are coloured in dark and light grey, respectively.

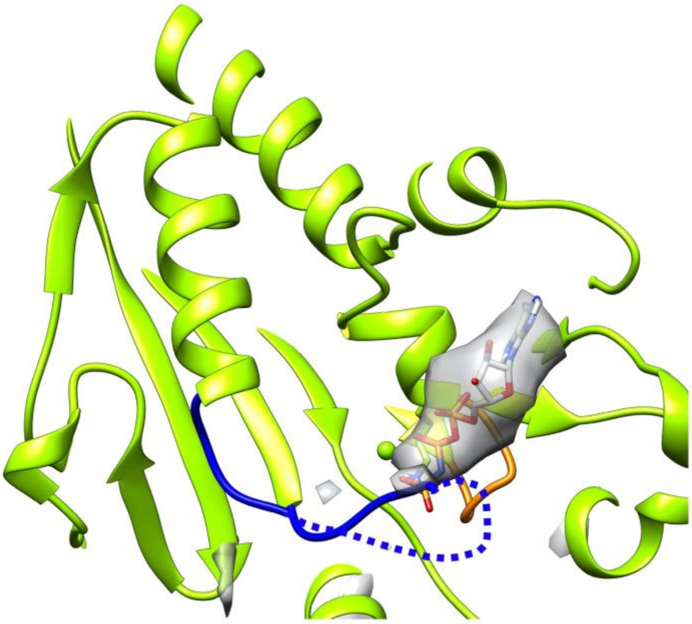

Supplementary Figure 7. AMPPNP binds to MT-bound *Pb*kinesin-8B-MD.
Difference density calculated between *Pb*kinesin-8B-MD-AMPPNP reconstruction and simulated 7 Å resolution density of the protein-only model. The difference map was calculated using Chimera “vop subtract” command. Density shown in transparent grey at threshold 0.0195 superimposed on the *Pb*kinesin-8B-MD-AMPPNP model, and corresponding with AMPPNP bound at the NBS. AMPPNP *Pb*kinesin-8B-MD model is coloured in light green, the P-loop is orange and flexible-appearing loop 9 is the dashed blue line.

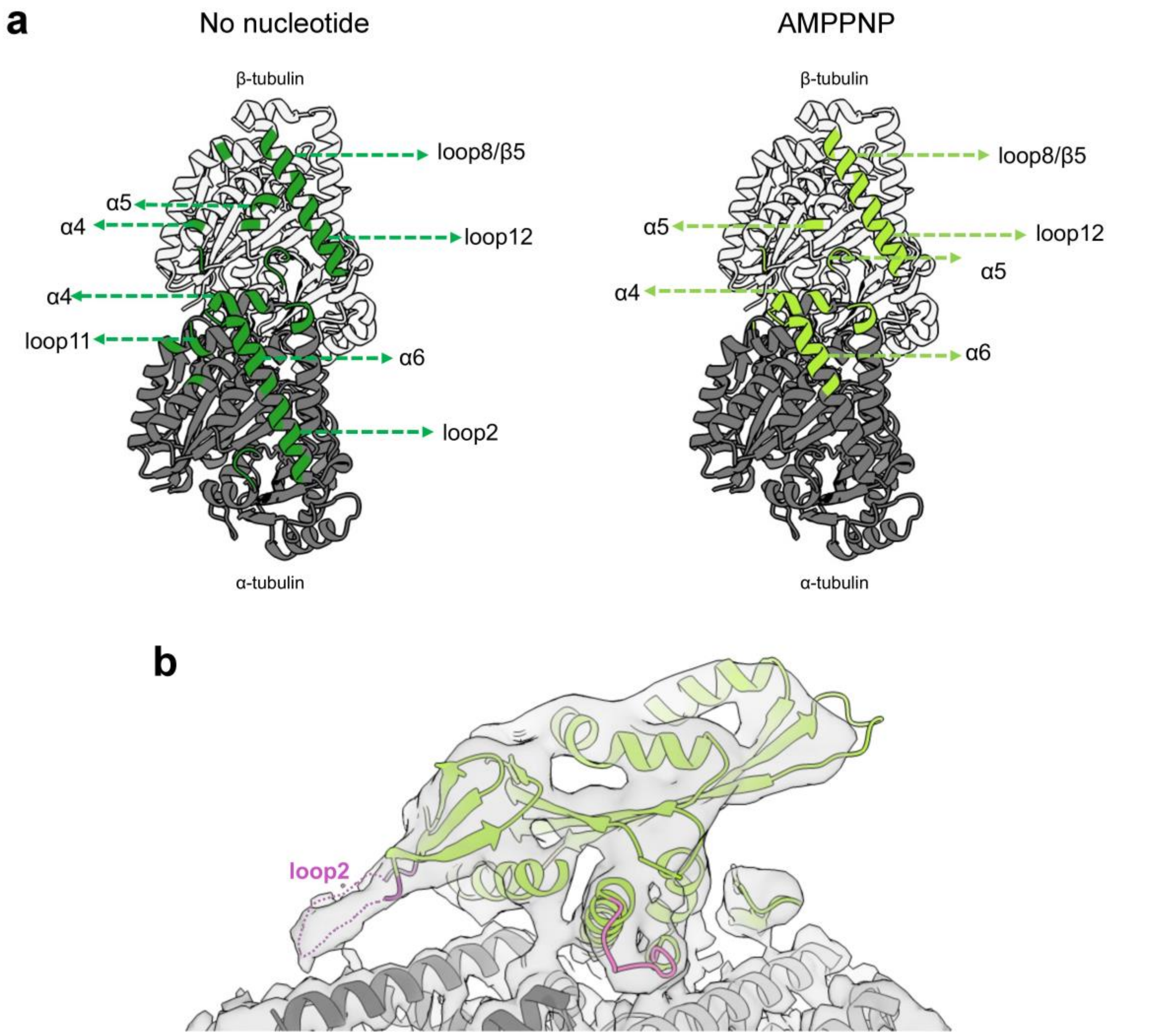

Supplementary Figure 8. Comparison of nucleotide-dependent *Pb*kinesin-8B-MD footprints on the MT surface.

a) Contacts formed between *Pb*kinesin-8B-MD and the MT in NN state and AMPPNP state are very similar. Left: MT footprint of *Pb*kinesin-8B-MD in NN state in dark green; dashed lines indicate contacting secondary structure elements in *Pb*kinesin-8B-MD. Tubulin residues <5Å distance from the bound motor is colored in dark green. Right: MT footprint of *Pb*kinesin-8B-MD in AMPPNP state in yellow green. α-tubulin are otherwise depicted in dark grey ribbon and β-tubulin in light grey ribbon.

b) View of the of MT-bound *Pb*kinesin-8B-MD in the AMPPNP state at threshold 0.0186 showing the contact formed between α-tubulin and loop2 (purple).

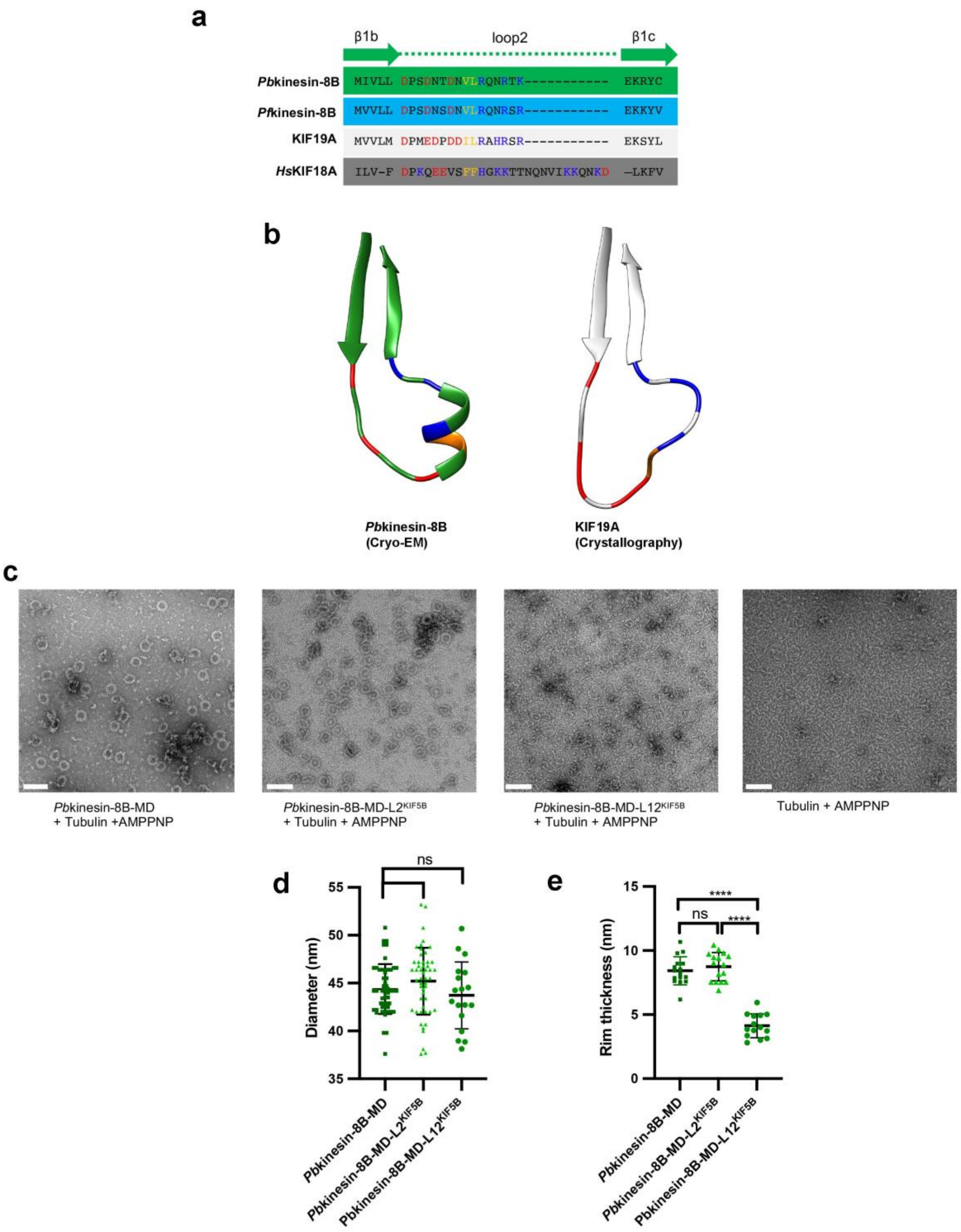

Supplementary Figure 9. Comparison of loop 2 of mammalian and malaria kinesin-8s, and characterisation of *Pb*kinesin-8B-MD loop 2 mutant-induced tubulin ring-like structures.

a) Sequence alignment of loop 2 from *Pb*kinesin-8B, *Pf*kinesin-8B, human KIF18A and mouse KIF19A. Positively charged residues are coloured blue, negatively charged residues are coloured red and hydrophobic residues are coloured orange.

b) Loop 2 of *Pb*kinesin-8B (MT-bound NN model) and KIF19A (PDB 5GSZ) are the same length but adopt different conformations.
c) Negative stain EM images showing the formation of tubulin rings by incubation of WT *Pb*kinesin-8B-MD, *Pb*kinesin-8B-MD-L2^{KIF5B} or *Pb*kinesin-8B-MD-L12^{KIF5B} with tubulin and AMPPNP; these structures do not form in the absence of *Pb*kinesin-8B. Scale bar: 100nm. The experiments were repeated three times.
d) The diameter of tubulin rings - measured in Fiji - formed by *Pb*kinesin-8B-MD-L2^{KIF5B} and *Pb*kinesin-8B-MD-L12^{KIF5B} is indistinguishable from those formed by WT *Pb*kinesin-8B-MD. Mean diameter of rings formed by WT *Pb*kinesin-8B-MD = 44.4 ± 2.6nm (mean ± SD, n = 53); mean diameter of rings formed by *Pb*kinesin-8B-MD-L2^{KIF5B} = 45.2 ± 3.5nm (mean ± SD, n = 51); mean diameter of rings formed by *Pb*kinesin-8B-MD-L12^{KIF5B} = 43.7 ± 3.5 nm (mean ± SD, n = 18); ns, not significant, p = 0.3161 (*Pb*Kinesin-8B-MD vs. *Pb*kinesin-8B-MD-L2^{KIF5B}) and 0.7302 (*Pb*kinesin-8B-MD vs. *Pb*kinesin-8B-MD-L12^{KIF5B}) by ordinary one-way ANOVA.
e) The thickness of the rim of the tubulin rings - measured in Fiji - formed by *Pb*kinesin-8B-MD-L12^{KIF5B} is statistically significantly different compared to those formed by WT *Pb*kinesin-8B-MD and *Pb*kinesin-8B-MD-L2^{KIF5B}. Mean thickness of rings formed by WT *Pb*kinesin-8B-MD = 8.4 ± 1.1 nm (mean ± SD, n = 16); mean thickness of rings formed by *Pb*kinesin-8B-MD-L2^{KIF5B} = 8.7 ± 1.1 nm (mean ± SD, n = 16); mean thickness of rings formed by *Pb*kinesin-8B-MD-L12^{KIF5B} = 4.1 ± 0.9 nm (mean ± SD, n = 14);****, p-values < 0.0001; ns, not significant, p = 0.6672 by ordinary one-way ANOVA.

a

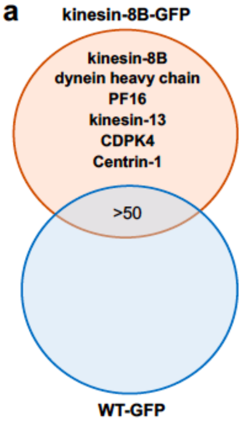

b

Identified Proteins	*P. berghei* gene ID	Molecular weight	Peptide numbers			
			kinesin-8B-GFP		WT-GFP	
			Exp1	Exp2	Exp1	Exp2
kinesin-8B	PBANKA_0202700	169 kDa	80	53	0	0
dynein heavy chain, putative	PBANKA_0416100	685 kDa	12	3	0	0
dynein heavy chain, putative	PBANKA_0925400	605 kDa	8	2	0	0
conserved Plasmodium protein, unknown function	PBANKA_1320100	93 kDa	5	13	0	0
kinesin-13, putative	PBANKA_1458300	117 kDa	3	2	0	0
armadillo repeat protein PF16	PBANKA_0917400	58 kDa	3	2	0	0
calcium-dependent protein kinase 4	PBANKA_0615200	61 kDa	3	2	0	0
flagellar outer arm dynein-associated protein, putative	PBANKA_0504300	12 kDa	3	2	0	0
gamete egress and sporozoite traversal protein	PBANKA_1312700	29 kDa	3	3	0	0
centrin-1, putative	PBANKA_0206300	20 kDa	1	1	0	0

Supplementary Figure 10. Interacting partners of *Pb*kinesin-8B-GFP in activated gametocytes identified by proteomics.

a) Venn diagram highlighting the major proteins pulled down by *Pb*kinesin-8B-GFP (orange) compared to GFP-only control (blue);

b) List of proteins pulled down with *Pb*kinesin-8B-GFP showing the number of unique peptides identified by mass spectrometry for the top candidates (n = 2 (Exp 1 and Exp2) for each condition).

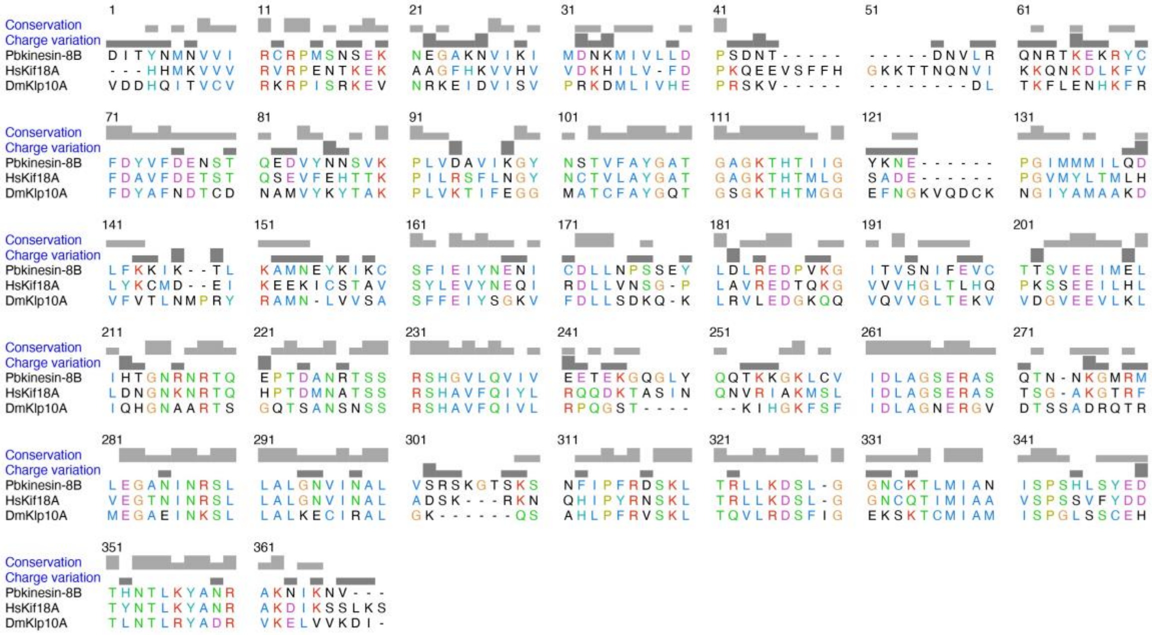

Supplementary Figure 11. Sequence alignments of *Pb*kinesin-8B, *Hs*Kif18A and *Dm*Klp10A motor domains.
Alignments were done with Clustal Omega. Residue colouring uses the Clustal X scheme.

References

1 Cook, A. D. *et al.* Cryo-EM structure of a microtubule-bound parasite kinesin motor and implications for its mechanism and inhibition. *J Biol Chem* **297**, 101063, doi:10.1016/j.jbc.2021.101063 (2021).

2 Cook, A. D., Manka, S. W., Wang, S., Moores, C. A. & Atherton, J. A microtubule RELION-based pipeline for cryo-EM image processing. *J Struct Biol* **209**, 107402, doi:10.1016/j.jsb.2019.10.004 (2020).
