# Supplementary material for: Mechanochemical tuning of a kinesin motor essential for malaria parasite transmission
Source: Nat Commun. 2022 Nov 16;13:6988. doi: 10.1038/s41467-022-34710-x (PMC9669022; doi:10.1038/s41467-022-34710-x)
Supplement: Supplementary file 3 — Description of Additional Supplementary Files [file 41467_2022_34710_MOESM3_ESM.pdf]

Description of Additional Supplementary Files

Supplementary Movie Legends

Supplementary Movie 1. Morph showing the structurally minimal response of MT-bound *Pb*kinesin-8B-MD around the nucleotide binding site on AMPPNP binding. Minimal structural response upon AMPPNP binding is shown by morphing cryo-EM density and fitted model between MT-bound *Pb*kinesin-8B-NN and *Pb*kinesin-8B-AMPPNP reconstructions using ChimeraX's 'volume morph' tool. The *Pb*kinesin-8B-NN fitted model is shown first and substituted for the *Pb*kinesin-8B-AMPPNP fitted model at the end of the density morph. No large global conformation changes were observed. Even with AMPPNP binding, the tips of both loop 9 and loop 11 are weak indicating the flexibility of these loops and the open state of the nucleotide binding site. Model morph (in grey) was generated using ChimeraX's 'morph' tool with two models (coloured) aligned on the tubulin dimer before morphing.

Supplementary Movie 2. Morph showing the structurally minimal response of MT-bound *Pb*kinesin-8B-MD around the neck linker region on AMPPNP binding. Minimal structural response upon AMPPNP binding is shown by morphing cryo-EM density and fitted model between MT-bound *Pb*kinesin-8B-NN and *Pb*kinesin-8B-AMPPNP reconstructions using ChimeraX's 'volume morph' tool. The *Pb*kinesin-8B-NN fitted model is shown first and substituted for the *Pb*kinesin-8B-AMPPNP fitted model at the end of the density morph. Upon AMPPNP binding, Neck linker density becomes weak and is clearly not docked onto the motor domain. Model morph (in grey) was generated using ChimeraX's 'morph' tool with two models (coloured) aligned on tubulin dimer density before morphing.

Supplementary Data. PCR primers used in this study.
