# Supplementary material for: Mechanochemical tuning of a kinesin motor essential for malaria parasite transmission
Source: Nat Commun. 2022 Nov 16;13:6988. doi: 10.1038/s41467-022-34710-x (PMC9669022; doi:10.1038/s41467-022-34710-x)
Supplement: Supplementary file 7 — Reporting Summary [file 41467_2022_34710_MOESM7_ESM.pdf]

nature portfolio

Corresponding author(s): Carolyn Moores
Last updated by author(s): Oct 28,2022

Reporting Summary

Nature Portfolio wishes to improve the reproducibility of the work that we publish. This form provides structure for consistency and transparency in reporting. For further information on Nature Portfolio policies, see our Editorial Policies and the Editorial Policy Checklist.

Statistics

For all statistical analyses, confirm that the following items are present in the figure legend, table legend, main text, or Methods section.

n/a	Confirmed	
☐	☒	The exact sample size (*n*) for each experimental group/condition, given as a discrete number and unit of measurement
☐	☒	A statement on whether measurements were taken from distinct samples or whether the same sample was measured repeatedly
☐	☒	The statistical test(s) used AND whether they are one- or two-sided<br>*Only common tests should be described solely by name; describe more complex techniques in the Methods section.*
☒	☐	A description of all covariates tested
☒	☐	A description of any assumptions or corrections, such as tests of normality and adjustment for multiple comparisons
☐	☒	A full description of the statistical parameters including central tendency (e.g. means) or other basic estimates (e.g. regression coefficient) AND variation (e.g. standard deviation) or associated estimates of uncertainty (e.g. confidence intervals)
☐	☒	For null hypothesis testing, the test statistic (e.g. *F*, *t*, *r*) with confidence intervals, effect sizes, degrees of freedom and *P* value noted<br>*Give P values as exact values whenever suitable.*
☒	☐	For Bayesian analysis, information on the choice of priors and Markov chain Monte Carlo settings
☒	☐	For hierarchical and complex designs, identification of the appropriate level for tests and full reporting of outcomes
☒	☐	Estimates of effect sizes (e.g. Cohen's *d*, Pearson's *r*), indicating how they were calculated

Our web collection on statistics for biologists contains articles on many of the points above.

Software and code

Policy information about availability of computer code

Data collection	MT- and tubulin-stimulated ATPase assay data was collected with SoftMax Pro 5 software. MT gliding assay and MT depolymerisation assay images were collected with the NIS-Elements AR Software (Nikon).Cryo-EM dataset of Pbkinesin-8B-MD NN was collected on Tecnai G2 Polara microscope with serial EM software. Cryo-EM dataset of Pbkinesin-8B-MD AMPPNP and Pfkinesin-8B-MD NN were collected on Titan Krios with EPU software (Thermo Fisher Scientific). Negative stain images were collected using Digital Micrograph™ (DM) software (Gatan).
Data analysis	MT gliding rate and MT depolymerisation rate were determined from kymographs using Fiji v2.1.0 software. Kcat and Km were determined using Microsoft Excel and Prism 9. The diameters and rim thickness of tubulin rings were measured in Fiji v2.1.0. Cryo-EM data were processed using MiRP pipeline (https://github.com/moores-lab/MiRP).CTFFIND4,MotionCor2,Relion v3.0 , EMAN2 v2.13, Cryosparc v2.11.0 and Python 2.7.5 were used during data processing. Models were built using MODELLER v9.23, Chimera v1.11.2,Coot 0.9.8.1 and Rosetta v3.7.Cryo-EM maps and models were displayed using Chimera X. Max QuantMax Quant (https://www.maxquant.org/) and Scaffold search engines were used for mass spectrometry data analysis. PlasmoDB (https://plasmodb.org/plasmo/app) was used for protein annotation.

For manuscripts utilizing custom algorithms or software that are central to the research but not yet described in published literature, software must be made available to editors and reviewers. We strongly encourage code deposition in a community repository (e.g. GitHub). See the Nature Portfolio guidelines for submitting code & software for further information.

Data

Policy information about availability of data

All manuscripts must include a data availability statement. This statement should provide the following information, where applicable:

- Accession codes, unique identifiers, or web links for publicly available datasets
- A description of any restrictions on data availability
- For clinical datasets or third party data, please ensure that the statement adheres to our policy

The MT-bound Pbkinesin-8B-MD_NN, Pbkinesin-8B-MD_AMPPNP and Pfkinesin-8B-MD_NN datasets have been deposited with the Electron Microscopy Public Image Archive85, deposition number EMPIAR-11115 [https://www.ebi.ac.uk/empiar/EMPIAR-11115], EMPIAR-11116 [https://www.ebi.ac.uk/empiar/EMPIAR-11116] and EMPIAR-11086 [https://www.ebi.ac.uk/empiar/EMPIAR-11086] respectively. The MT-bound Pbkinesin-8B-MD_NN, Pbkinesin-8B-MD_AMPPNP and Pfkinesin-8B-MD_NN reconstructions have been deposited with the Electron Microscopy Data Bank86, deposition number EMD-14459 [https://www.ebi.ac.uk/pdbe/entry/emdb/EMD-14459], EMD-14460 [https://www.ebi.ac.uk/pdbe/entry/emdb/EMD-14460] and EMD-14461 [https://www.ebi.ac.uk/pdbe/entry/emdb/EMD-14461] respectively. The molecular models of MT-bound Pbkinesin-8B-MD_NN, Pbkinesin-8B-MD_AMPPNP and Pfkinesin-8B-MD_NN have been deposited with the Worldwide Protein Data Bank87, deposition number 7Z2A [http://doi.org/10.2210/pdb7z2a/pdb], 7Z2B [http://org.doi/10.2210/pdb7z2b/pdb] and 7Z2C [http://doi.org/10.2210/pdb7z2c/pdb] respectively. PDB models used for structure comparison and model building can be found with the following accessible link: PDB 6OJQ [http://doi.org/10.2210/pdb6ojq/pdb],PDB 5GSZ [http://doi.org/10.2210/pdb5gsz/pdb] 21, PDB 4LNU [http://doi.org/10.2210/pdb4lnu/pdb]35, PDB 3HQD [http://doi.org/10.2210/pdb3hqd/pdb]39, and PDB 4OZQ [http://doi.org/10.2210/pdb4ozq/pdb]72 . Proteomics data generated in this study have been deposited in the ProteomeXchange Consortium via the PRIDE partner repository with the dataset identifier PXD037474 [http://proteomecentral.proteomexchange.org/cgi/GetDataset?ID=PXD037474]
Source data are provided with this paper.

Human research participants

Policy information about studies involving human research participants and Sex and Gender in Research.

Reporting on sex and gender	n/a
Population characteristics	n/a
Recruitment	n/a
Ethics oversight	n/a

Note that full information on the approval of the study protocol must also be provided in the manuscript.

Field-specific reporting

Please select the one below that is the best fit for your research. If you are not sure, read the appropriate sections before making your selection.

☒ Life sciences ☐ Behavioural & social sciences ☐ Ecological, evolutionary & environmental sciences

For a reference copy of the document with all sections, see nature.com/documents/nr-reporting-summary-flat.pdf

Life sciences study design

All studies must disclose on these points even when the disclosure is negative.

Sample size	The sample size for cryo-EM study was determined based on previous similar studies. Hundreds or thousands movies are needed to obtain a high resolution reconstruction(<5Å) to ensure confidence in conclusions drawn from the study. For the Pbkinesin-8B-MD NN cryo-EM dataset, 329 movies were collected. For Pbkinesin-8B-MD AMPPNP cryo-EM dataset, 1026 movies were collected. The Pfkinesin-8B-MD NN cryo-EM dataset consisted of 4075 movies.For the Pbkinesin-8B-MD-tubulin cryo-EM dataset, 8148 movies were collected. Dozens or hundreds individual MTs from different microscopic fields of view were tracked for MT gliding and depolymerisation assay for each protein to ensure most trackable MTs are included in the analysis and the datasets represent the overall population of microtubules in each condition.
Data exclusions	Cryo-EM images were selected in a non-biased manner by Relion 3.0 subset selection job using well defined criteria (max resolution,defocus etc.). MTs with 14 protofilaments were selected by Relion 3.0 supervised 3D classification job for data processing, and the particle numbers for the final 3D reconstructions are indicated in Table 1.
Replication	All measurements for ATPase activity, microtubule gliding and microtubule depolymerization activity were done in triplicate (three independent experiments per protein prep). All attempts at replication of these experiments were successful. For negative stain data and Cryo-EM datasets, similar images were obtained from 2-3 preliminary test datasets. Data for 3D reconstruction and data analysis were collected from one EM grid for each sample. Proteomics analyses were performed on two separate parasite samples.

Randomization	During cryoEM data processing, each dataset was randomly split to two half for calculating gold-standard Fourier Shell Correlation (FSC). Randomization was not relevant in the other experiments because of the nature of the study design. The experiments involving animals related to parasites and not the mice.
Blinding	Blinding is not feasible for our experiments because of the nature of our study design.

Reporting for specific materials, systems and methods

We require information from authors about some types of materials, experimental systems and methods used in many studies. Here, indicate whether each material, system or method listed is relevant to your study. If you are not sure if a list item applies to your research, read the appropriate section before selecting a response.

Materials & experimental systems

n/a	Involved in the study
☒	☐ Antibodies
☒	☐ Eukaryotic cell lines
☒	☐ Palaeontology and archaeology
☐	☒ Animals and other organisms
☒	☐ Clinical data
☒	☐ Dual use research of concern

Methods

n/a	Involved in the study
☒	☐ ChIP-seq
☒	☐ Flow cytometry
☒	☐ MRI-based neuroimaging

Animals and other research organisms

Policy information about studies involving animals; ARRIVE guidelines recommended for reporting animal research, and Sex and Gender in Research

Laboratory animals	Six- to eight-week-old female CD1 outbred mice were used for all experiments
Wild animals	The study did not involve wild animals
Reporting on sex	Only female mice were used to prepare gametocyte material with no consideration of sex differences because the focus of the study was the parasites and not the host mice.
Field-collected samples	The study did not involve samples collected from the field
Ethics oversight	The animal work required to prepare gametocyte material passed an ethical review process at Nottingham University and was approved by the United Kingdom Home Office.

Note that full information on the approval of the study protocol must also be provided in the manuscript.
